# Taking Stock of the *Drosophila* Research Ecosystem

**DOI:** 10.1534/genetics.117.202390

**Published:** 2017-06-29

**Authors:** David Bilder, Kenneth D. Irvine

**Affiliations:** * Department of Molecular and Cell Biology, University of California-Berkeley, California 94720; † Waksman Institute, Rutgers University, Piscataway, New Jersey 08854; ‡ Department of Molecular Biology and Biochemistry, Rutgers University, Piscataway, New Jersey 08854

**Keywords:** *Drosophila*, advocacy, community, genetics, model organism

## Abstract

With a century-old history of fundamental discoveries, the fruit fly has long been a favored experimental organism for a wide range of scientific inquiries. But *Drosophila* is not a “legacy” model organism; technical and intellectual innovations continue to revitalize fly research and drive advances in our understanding of conserved mechanisms of animal biology. Here, we provide an overview of this “ecosystem” and discuss how to address emerging challenges to ensure its continued productivity. *Drosophila* researchers are fortunate to have a sophisticated and ever-growing toolkit for the analysis of gene function. Access to these tools depends upon continued support for both physical and informational resources. Uncertainty regarding stable support for bioinformatic databases is a particular concern, at a time when there is the need to make the vast knowledge of functional biology provided by this model animal accessible to scientists studying other organisms. Communication and advocacy efforts will promote appreciation of the value of the fly in delivering biomedically important insights. Well-tended traditions of large-scale tool development, open sharing of reagents, and community engagement provide a strong basis for coordinated and proactive initiatives to improve the fly research ecosystem. Overall, there has never been a better time to be a fly pusher.

FOR over a century, the fruit fly *Drosophila melanogaster* has been a leading model organism for investigating the fundamental biology of animals (Box 1). Its contributions range from elucidating basic principles of heredity and genetics (exemplified by the Nobel Prizes awarded to T. H. Morgan and H. J. Muller), to uncovering the phylogenetically conserved components with which all animals develop (exemplified by the Nobel prizes awarded to E. B. Lewis, C. Nusslein-Volhard, and E. Wieschaus), to revealing molecular mechanisms of direct relevance to human immunity and health (exemplified by the Nobel Prize awarded to J. Hoffman). The fly continues to be at the frontiers of both foundational research, such as how neural circuits mediate complex behaviors, and biomedical impact, such as efficient modeling of human diseases. Flies are also central to insect management initiatives for vector-disseminated diseases and agriculturally important pollinators and pests.

Box 1 **A few examples of fundamental discoveries pioneered in *Drosophila***

Fruit fly research for over a century has led to breakthroughs that form the basis of our understanding of biology of all animals, including humans. A handful of these textbook discoveries are listed below, others are listed in Rubin and Lewis (2000), Bellen *et al.* (2010), and Wangler *et al.* (2015).

The relationship of genes to chromosomes and the mechanism of inheritance (Nobel 1933).
Radiation as a mutagenic agent (Nobel 1946).
Delineation of many conserved intracellular signaling pathways, including Notch, Hedgehog, and Wnt (Nobel 1995).
The deep conservation of animal development/biology, exemplified by Hox genes (Nobel 1995).
Toll-like receptors as the key regulators of innate immunity (Nobel 2011).
Identification of key functional components of growth regulatory pathways, including Ras and Hippo.
Genetic identification of chromatin regulators.
Mechanisms of stem cell niche activity.
The molecular nature of neural conduction, via Shaker and TRP channels.
The molecular basis of circadian rhythms.
Genetic approaches to analyzing animal behavior; neurogenetics.

Despite these outsized impacts, recent trends in funding for fly work, a perceived devaluation of fundamental research relative to translational research, and changes in National Institutes of Health (NIH) support for community infrastructure including model organism databases (MODs), have raised the specter of erosion to the *Drosophila* research powerhouse. At the same time, the growing numbers of *Drosophila* researchers, in countries such as China and India, emphasizes the need to communicate about large-scale resource initiatives for maximum efficiency.

*Drosophila* scientists have a long-standing organizational body, the Fly Board, that is charged with coordinating the community, overseeing shared resources, and advocating for fly-centered research. With these issues in mind, we—the 2015 and 2016 Presidents of this body—convened a workshop of community representatives, resource generators, and infrastructure leaders from around the world. This meeting, hosted by the Howard Hughes Medical Institute (HHMI) at its Janelia Research Campus, took place in February 2016. Sessions were devoted to reviewing *Drosophila* research support and infrastructure, discussing how to maintain its health and productivity, and envisioning where it should grow in the future. Here, we discuss the current state of the “*Drosophila* research ecosystem,” focusing on resources and community, and its prospects for extending the fly’s long history at the leading edge of discovery.

## The “Fly Worker Ethos”

What factors have placed flies at the forefront of research for so long? In addition to the many biological advantages of *Drosophila* (Box 2), fly work has benefited from a strong community. A distinctive feature of the *Drosophila* community is the commitment of researchers to a shared set of principles known as the “fly worker ethos.” These principles were set in practice by the Morgan group, and as described by Kohler (1994), include:

unconstrained sharing of published reagents;
generation and widespread distribution of community resources;
innovation and dissemination of innovative genetic tools;
open communication alongside healthy competition.

Box 2 **Some strengths of *Drosophila* as a research organism**

A unique combination of strengths have helped to establish *Drosophila* as a leading animal model for biomedical research. These include:

A simple genome and short generation time, facilitating rapid genetic analysis as well as evolutionary studies.
Low animal costs compared to vertebrate models.
A wealth of information accumulated over a century of research on its genetics, genomics, development, physiology,  ecology, and evolution.
A vigorous and vibrant community with a tradition of pioneering and sharing resources and tools.
A complex yet easily manipulated animal suited for a broad range of *in vivo* investigations, including organ  development, signaling and gene regulatory networks, transcriptional regulation, epigenetics, and the genetic  basis of complex traits.
A remarkably conserved physiology useful for modeling innumerable processes, including identification and  characterization of human disease genes.
A sophisticated nervous system allowing studies of neural function across scales from molecules to behaviors.
An extensive and accessible toolkit that provides diverse strategies for manipulation and visualization of gene function,  including unprecedented spatiotemporal control over genetic manipulations.
An unparalleled collection of community-established resources including *Drosophila* and molecular stock centers, and  the pioneering bioinformatics resource FlyBase.

This ethos places a strong emphasis on developing tools that are of general utility for leveraging the fly’s powerful genetics. Importantly, Kohler emphasizes that the ethos is in part a moral code, but is also “enlightened self-interest”: the more that individuals contribute to the collective good, the stronger the overall impact of fly work is. In a more recent formulation (Rubin 2015): “[Fly workers’] fortunes rise and fall together, based not on which of us publishes some result first, but on how the value of fly research *as a whole* is perceived.”

Thus, tools, resources, and their open distribution have long lay at the heart of the fly community. The initiative of resource creators, along with the foresight of funding agencies to support their work, has led to the development of numerous foundational tools, such as the GAL4/UAS and FLP/FRT methods for controlling gene expression, and resource efforts including the Berkeley *Drosophila* Genome Project (BDGP) and ModEncode. Indeed, the *Drosophila* database FlyBase pioneered what MODs could be, well before their value as a “data resource” was widely appreciated. These advances that were developed or perfected in flies have served as paradigms and inspiration for analogs in other model organisms. Excellent reviews of resources available to fly researchers have been published recently (*e.g.*, Cook *et al.* 2010; Mohr *et al.* 2014; Marygold *et al.* 2016). A brief description of these—including new ones that are emerging—is provided below.

## Physical and Data Resources

### Resources for analysis of genes and phenotypes *in vivo*

A powerful advantage of *Drosophila* as an experimental system lies in its wide repertoire of available genetic manipulations. Establishing a complete set of reagents to manipulate each of the ∼14,000 protein-coding genes in the genome with both loss-of-function and gain-of-function approaches, as well as to identify the localization of each protein in the organism and within its cells, are long-term goals of the fly community.

#### Genetic mutations:

Loss-of-function alleles have currently been generated for 56% of genes, including 77% of the genes orthologous to human genes (N. Brown FlyBase Consortium, personal communication). Of these, 21% have alleles induced by chemical or X-ray mutagenesis, while ∼65% have only transposon insertion alleles; some may not be functionally null. Clustered regularly interspaced short palindromic repeats (CRISPR)-based efforts to generate new null alleles will increase this number in the immediate future, and these may have fewer unlinked genetic alterations than alleles generated by random mutagenesis. Not all useful alleles are available via public stock centers; the Bloomington *Drosophila* Stock Center (BDSC) actively encourages submission of well-documented null alleles in genes that are not represented in their collection.

In addition to mutations generated by individual investigators, the Gene Disruption Project has generated a large-scale collection of transposable element insertions (Bellen *et al.* 2011). The vectors used for insertional mutagenesis have become more sophisticated, enabling not only simple truncation of an open reading frame, but also exon trapping to generate loss-of-function alleles, as well as protein tagging (below) (Venken *et al.* 2011; Nagarkar-Jaiswal *et al.* 2015). Past efforts took advantage of random insertions, but new initiatives will use CRISPR/Cas9-mediated site-specific targeting to extend the *Drosophila* mutant kit (Zhang *et al.* 2014; Diao *et al.* 2015).

#### Targeted gene manipulation:

The ability to manipulate gene expression or activity with tight spatial and temporal control is a tremendous strength of the fly. Much of this power comes through exploiting and refining the Gal4/UAS system and its derivatives (Brand and Perrimon 1993; del Valle Rodríguez *et al.* 2011), to drive expression of wild-type or variant alleles in overexpression analysis, and targeted knockout or knockdown of gene expression for loss-of-function analysis.

##### Tissue-specific drivers:

There exist ∼15,000 lines in which *Drosophila* *cis*-regulatory sequences can be used to drive expression of paired “responder” lines through a simple genetic cross (FlyBase Consortium, personal communication). Most of these “drivers” are based on the GAL4 transcriptional activator, but a growing number use “orthogonal” activation systems such as QF and LexA (Lai and Lee 2006; Potter *et al.* 2010). Expression patterns of many have been documented in various tissues, including the embryo, imaginal discs, and brain (Jenett *et al.* 2012; Jory *et al.* 2012; Manning *et al.* 2012). Together with activator or inhibitor variants that can be controlled by temperature or drugs, or by “intersectional” crosses, these enable transgenes to be expressed in an enormous variety of temporal and spatial patterns (del Valle Rodríguez *et al.* 2011). Recent collections of stocks insert T2A-GAL4 sequences into introns for in-frame splicing, generating Gal4 lines that closely follow endogenous gene expression patterns and hence are well-suited for gene rescue or gene replacement approaches (Diao *et al.* 2015).

##### Gene product knockdown:

RNA interference (RNAi) constructs, drivable by the GAL4/UAS system, are now available for ∼98% of fly genes, with multiple lines available for 90% (J. Zirin FlyBase Consortium, personal communication). Because the efficacy of an RNAi construct cannot be predicted *a priori*, with perhaps 15% of them being ineffective and some inducing off-target effects, a community annotation website called RSVP has been created to report the efficacy of a given construct (Perkins *et al.* 2015). Increased participation in RSVP would save *Drosophila* researchers a good deal of time and effort.

##### Targeted expression constructs:

UAS lines, generated either by random transposon insertion (“EP lines”) or by gene-by-gene transgenesis, are currently available for over 4500 genes, as well as ∼180 miRNAs (Rorth 1996; Toba *et al.* 1999; Bellen *et al.* 2011; Bejarano *et al.* 2012) (N. Brown FlyBase Consortium, personal communication). Such lines are often used to determine phenotypes induced by misexpression or overexpression of a gene product of interest, and to express alternate alleles to compare function. Many existing lines incorporate epitope tags that allow the localization, immunoprecipitation, and in some cases live imaging of the protein products. Creation of a UAS-driven collection of tagged human cDNAs, homologous to fly genes, is currently in progress. By expressing wild-type and mutant variants, these can be used to help understand the biological functions of human disease genes, and the consequences of specific mutations (Bellen and Yamamoto 2015).

#### CRISPR resources:

CRISPR/Cas9 has revolutionized the genetic manipulation of flies, as it has for other organisms. Protocols and reagents have been developed that make CRISPR-mediated mutagenesis straightforward for all fly labs (Gratz *et al.* 2015) (http://flycrispr.molbio.wisc.edu; http://www.crisprflydesign.org). Large libraries of gRNAs are being created to target CRISPR constructs to most *Drosophila* genes *in vivo*. Beyond genome editing, these can be used in combination with modified Cas9 proteins to effectively activate gene expression (CRISPRa) (Lin *et al.* 2015). CRISPRa approaches are complementary to UAS-driven approaches as they allow more physiological expression levels, and can express multiple isoforms of a gene. Tissue-specific expression of catalytically active Cas9 with gRNAs can allow the generation of genetically mosaic animals with biallelic mutation of a given gene (Port *et al.* 2014; Xue *et al.* 2014). This approach complements FLP/FRT recombination-based mosaic approaches; both create truly null alleles that evade concerns about partial gene product inhibition that are associated with RNAi-based approaches.

#### Protein localization:

The traditional approach to visualize the expression pattern and subcellular localization of proteins utilizes antibodies. While *in vivo* epitope tagging projects (described below) provide many advantages, these are not always practical with complex fly genetics, so improved quality and quantity of antibodies remains a priority for the community. The pool of available antibodies to *Drosophila* proteins is still fairly limited, and is estimated to be ∼500 (H. Bellen, personal communication). The Developmental Studies Hybridoma Bank distributes ∼250 monoclonal antibodies made to *Drosophila* antigens, but others are polyclonal antibodies distributed by individual labs and are a nonrenewable resource. Knowledge of commercial mammalian antibodies that cross-react to *Drosophila* proteins is scattered; an initiative to collect this information is underway. A high-throughput approach based on phage-display of synthetic antibodies has been successfully applied to the generation of antibodies against *Drosophila* RNA-binding proteins (Na *et al.* 2016).

##### Fluorescently-tagged proteins:

Tagging proteins at endogenous loci through genetic engineering provides an alternative to antibody production, allowing not only tissue-wide and subcellular information, but also reliable immunoprecipitation, western blotting, and often live imaging. Large-scale approaches have substantially increased the availability of tagged proteins. An initial collection based on random insertion of a transposable GFP-encoding exon (“protein traps”) (Morin *et al.* 2001; Buszczak *et al.* 2007; Quinones-Coello *et al.* 2007; Lowe *et al.* 2014) has been succeeded recently by other approaches. In one, intronic insertions of either the *Minos*-mediated integration cassette (“MiMIC”) transposon or a gRNA-targeted CRISPR-mediated integration cassette (‘CRIMIC’) construct, which both contain an easily exchangeable ϕC31-based cassette, can be used for tagging with any protein sequence (Venken *et al.* 2011; Nagarkar-Jaiswal *et al.* 2015). In another, genomic P[acman] BACs or fosmids manipulated to tag protein-coding regions have been inserted into transgenic flies (Ejsmont *et al.* 2009; Venken *et al.* 2009). To date, ∼2000 fly stocks carrying proteins tagged by the above strategies have been made publicly available. In addition, 10,000 more tagged fosmids have been created and can be purchased ready for transformation (Sarov *et al.* 2016).

GFP tagging of endogenous loci also enables the use of a novel protein depletion approach called deGradFP (degrade GFP) (Caussinus *et al.* 2011). deGradFP involves degradation of proteins by nanobody-mediated ubiquitination of the GFP tag. The acute protein loss induced by this technique avoids issues of gene product perdurance associated with other loss-of-function methods.

### *Drosophila* stock and resource centers

Publicly available collections of the resources above are a critical pillar of *Drosophila* research. Fly stocks are the most frequently used of these resources. The *Drosophila* community currently enjoys several stock centers in the US, EU, Japan, India, and China; each provides fee-based public access to some of the ∼80,000 *Drosophila* variants currently maintained, as well as to related species. These centers provide easy accessibility, stably maintained reagents, and economies of scale; each serves unique needs by maintaining distinct collections as well as providing a local source for high-demand stocks.

To highlight just one example, the BDSC stocks nearly 60,000 unique genotypes, and ships out over 200,000 stocks each year to >3000 users (Box 3). Capacity at the BDSC in its current environs at Indiana University is not infinite, but it presently can accommodate perhaps 80,000 stocks. In addition to maintaining and distributing stocks, the BDSC also provides information on their use (*e.g.*, chromosome mechanics) and occasionally assists in stock generation (*e.g.*, molecularly defined chromosomal deficiencies). As public resources important for scientific progress, stock centers need the security and stability provided by continued public investment and oversight. Support for the BDSC comes partly from NIH grants, but 75% is covered by user fees. By distributing well-defined and stably maintained reagents, the BDSC and the other stock centers play an essential role in enabling researchers to build on the progress of their colleagues, and aid in establishing reproducibility. This effectively fulfills NIH mandates in a reliable and cost-effective way.

Box 3 **The BDSC**

The BDSC, hosted by Indiana University and in its 30th year of operation, maintains the largest public collection of *Drosophila* stocks and epitomizes the resource infrastructure that supports fly research. Stocks are distributed through a straightforward web-based ordering system and mailed out weekly throughout the year. A few statistics of BDSC resources and usage are given below:

59,000 stocks with 62,800 unique genetic components (alleles, transgene insertions, aberrations, etc.).
2600 stocks were donated in 2016 by 98 donors at 63 organizations.
6800 registered users in 3300 laboratories in 66 countries.
217,000 samples distributed in 13,500 shipments in 2016.
Fees can average as little as $4/stock.

The vigor of *Drosophila* research and the success of stock centers are deeply connected. Stock centers can build relevant and useful collections only when researchers donate important stocks for widespread distribution. Likewise, stock centers can cover their operational expenses only with robust use of stocks by researchers. In general, existing stock centers are in reasonably good financial condition, and worldwide stock capacity is expanding, but sustainability has become an issue for stock centers with smaller user bases. This is particularly true for centers distributing non-*melanogaster* *Drosophila* species, which are immensely valuable for ecological and evolutionary studies, but whose overall demand is relatively low. Stock charges vary from center to center due to different funding structures, and high prices can be a barrier to usage of certain collections. Finally, import regulations in several regions are hampered by burdensome procedures that do not take into account the innocuousness of model organisms and create impediments to the international exchange of stocks.

Unlike the strains of many other genetic model organisms, *Drosophila* strains have to be maintained as continuous living cultures. The development of robust methods for long-term preservation would provide more options for maintaining and distributing strains, allow the preservation of important but rarely used strains, prevent the accumulation of mutations associated with long-term culture, and help to secure genetic resources from disaster. Past efforts at cryopreservation have not worked well enough to be relied on for maintaining stock collections, but encouraging advances have been made on several technical fronts, as discussed at a recent NIH-sponsored workshop. A robust and efficient method would undoubtedly be a boon to the entire community.

#### Screening centers:

The utility of cell-based RNAi screens can be exploited by resources from two sites, the *Drosophila* RNAi Screening Center in Boston or the Deutsches Krebsforschungszentrum in Heidelberg (Horn *et al.* 2010; Hu *et al.* 2017). Each site distributes its own RNAi library; each site (as well as other sites in New York and Sheffield) also hosts visitors for screening using existing high-throughput facilities. These facilities can also support drug screening in cells, and reagents for CRISPR-Cas9 based screens are being developed. Whole-animal drug screening platforms have also been successfully used by several groups, and broader availability of drug screening would complement the increasing interest in fly models of human disease.

#### Molecular resources:

Many of the fruits of the decades-long BDGP are available at the *Drosophila* Genomics Resource Center (DGRC) or commercial distributors such as Addgene. This includes cDNA libraries and individual clones, including the sequence-confirmed and full-length “Gold” collection, as well as ORF collections in both Gateway and loxP-containing donor vectors (Stapleton *et al.* 2002) that have been transferred to expression vectors for tissue culture and animal transgenics (Guruharsha *et al.* 2011). Commonly used plasmids, including those for CRISPR engineering, can also be obtained. DGRC further distributes a collection of ∼180 *Drosophila*-derived cell lines (Cherbas and Gong 2014), in which interest is expanding due to their utility in high-throughput screens and ability to be modified using CRISPR and ϕC31 recombination-mediated transformation.

### Bioinformatic resources

Databases that provide access to the accumulated knowledge and resources generated by *Drosophila* research play an indispensable role in facilitating *Drosophila* research, so their maintenance and enhancement is a major community concern, especially in the light of potential changes to funding for MODs under consideration at NIH.

The central repository of information for *Drosophila* research is FlyBase (Gramates *et al.* 2017), which collects, curates, and links to diverse sources of information, including genes, their products and phenotypes, the genomics, development, and physiology of *Drosophila*, and accumulated reagents and data sets. This storehouse of *Drosophila* research knowledge is used daily by many in the community to interpret new data and to generate new hypotheses; it receives over 1,200,000 page views/month.

#### Curation:

More than 2400 articles about *Drosophila* are published annually. Their curation through FlyBase is essential to making the results that they describe accessible beyond an individual article, and integrated with the vast knowledge of fly biology. FlyBase curation allows these results to be linked through diverse routes, whether it be starting from the published literature or from a gene, organ, behavior, or network of interest. When compared to the time required for each individual scientist to separately chase down or discover relevant data, high-quality curation is extremely cost-effective.

Curation requires a substantial, ongoing effort, and endeavors to aid FlyBase’s professional curators with community input are underway. Community experts now contribute to short summaries of gene function. Almost 50% of authors aid in annotating reagents used in their publications through the “Fast Track Your Paper” tool. A new initiative to expand such annotation to each published manuscript is in progress. FlyBase has helped Cell Press and *GENETICS* and *G3* to develop the “STAR Methods” table and has created a standardized “Author Reagents Table.” It is hoped that other journals will adopt this requirement, which helps to fulfill NIH requirements for rigor and reproducibility and reagent validation. This type of active participation by the *Drosophila* community can play an important role in maintaining high-quality curation and reducing the burden on FlyBase support as the literature continues to expand. Nevertheless, there will be a continuing need for professional curation as well.

#### Integration:

Integration of data, both within FlyBase and across other MODs, are essential goals, but present significant challenges. *Drosophila* researchers are continually generating new data sets, which need to be linked with FlyBase to maximize their accessibility and utility to *Drosophila* researchers. To give just one example, exciting advances are being made in preparing anatomical and gene expression atlases, but the size and complexity of image data sets pose particular challenges, and most currently operate as stand-alone databases. The utility of crowd-sourced annotation of image data sets is substantial, and continued development and distribution of annotation tools should be encouraged.

To fully capitalize on the wealth of functional genetic information that decades of *Drosophila* research has generated, this information must be accessible to researchers studying other organisms. One of the most important priorities for the future is to effectively link FlyBase to other MODs, including databases such as Online Mendelian Inheritance in Man, which focus on human genes and human disease. Initial efforts to address this include the web resources MARRVEL.org and Gene2Function.org. Distinct databases have developed different ways of organizing and presenting data, but tighter integration will be essential to fully realize the impact that discoveries in model organisms can have on biological understanding and human health. In any such effort, it will be important to find ways to preserve the essential data contained within FlyBase that does not have parallels in other organism databases. This goal is being actively pursued by FlyBase and other MODs through the Alliance for Genome Resources.

#### Accessibility:

The ever-increasing volume of information provided by MODs creates challenges in displaying it so that key information is readily accessible, and readable by both humans and machines. Data are primarily organized by gene within FlyBase, but the ability to access data from different perspectives, such as by pathway, anatomy, or disease, is also important. FlyBase currently has a detailed Help section that includes video tutorials to aid new users in finding the information they need, but there was also broad agreement that an emphasis for the future should be to make *Drosophila* data sets more accessible to non-*Drosophila* researchers, such as researchers working with other model organisms, or clinicians working on human disease genes.

#### Sustainability:

The uncertainty regarding future funding of MODs has created concerns about the sustainability of FlyBase and other databases. Visionary early support from NIH made FlyBase and other MODs possible, and ongoing support of bioinformatics resources has fueled countless discoveries. It makes no sense for funding agencies to support biomedical research, and then allow the resulting discoveries to be lost or rendered inaccessible because of inadequate support for database infrastructure, especially when this support represents only a tiny fraction of overall research support. However, there has been real concern that this could happen. Community leaders have thus been actively discussing with NIH and other funders the essential roles that databases provide in collecting, preserving, and presenting scientific discoveries so that they can provide a foundation for continuing future discoveries. They have also emphasized the value of the diverse types of information currently maintained by *Drosophila* databases. Currently, 91% of public funding for FlyBase comes from the NIH National Human Genome Research Institute (NHGRI), with 9% from the Medical Research Council and the Wellcome Trust in the UK. However, Flybase users span the globe, and within the US include the ∼33% of NIH *Drosophila* grantees supported by the National Institute of General Medical Sciences and the ∼20% supported by the National Institute of Neurological Disorders and Stroke (NIH RePORTER, 2014–15). Increased support for MODs from sources other than the NHGRI, including within the international community, could help to maintain open access to FlyBase.

## Community and Advocacy

### Who is the fly community?

The tools described above would not be possible if there were not a strong community of researchers behind them. However, defining current community members is a challenge. Available data allow us to make some rough guesses of the size of the fly research community, albeit biased toward US-based researchers. Perhaps the best estimate comes from recent usage at BDSC, which likely reflects active working groups. By this measure, >3000 individual groups currently exist worldwide. This does not include overseas groups that do not order from BDSC; it also does not distinguish research labs from those who use flies only for teaching. Nevertheless, when all data (meeting attendance, mailing lists, and other stock centers) are considered, an estimate of >6000 fly workers worldwide seems conservative.

A contact list of active fly workers is desirable for a number of reasons. It would enable dissemination of information about new resources, allow Resource Centers and the *Drosophila* Board to solicit representative feedback on community needs, and could mobilize fly workers for important advocacy issues. Because of the advantages of such a comprehensive list, the Fly Board is currently reaching out to register PIs through active solicitation. Non-PIs can continue to join through the existing “Fly Person” registration.

### Community engagement

The most frequent locus of fly researcher engagement is FlyBase. Primarily a data resource, FlyBase has recently redesigned its site to emphasize hosted links relevant to community interests. In the past, web-based discussion boards such as bionet.drosophila existed for fly workers, to request technical advice, search for reagents, or advertise fly worker positions. A desire for a forum continues to exist; one possibility is the growing community website drosophilaresearch.org.

Other strategies to increase community involvement are available. Social media referencing *Drosophila* research, including the Twitter hashtag #drosophila and account @fly_papers, are active but would benefit from broader awareness. The Fly Board now encourages a more active role for regional representatives via committee service. The addition of a new Trainee representative will allow the Board to be responsive to postdoc and graduate student input. Supporting the development of new fly PIs is a critical goal. This year’s *Drosophila* Research Conference featured a session devoted to welcoming these PIs and networking them with established researchers. We envision this as a springboard to other initiatives, including mentoring relationships and perhaps a young investigators’ network building on the European FlyJEDI (Junior European *Drosophila* Investigators) network.

Barriers for newcomers to join the fly community should be lowered. For researchers who want to add flies to their research program, “boot camp”-style coursework is an ideal entry. The Wellcome Genome Institute hosts the *Drosophila* Genetics and Genomics course biennially in the UK; Cold Spring Harbor Laboratory runs a *Drosophila* Neurobiology class each year. More frequent coursework at additional sites would be welcome. Outside of formal coursework, an outstanding introductory training package has been created and published in *G3* (Roote and Prokop 2013); a recent Primer published in *GENETICS* provides a nice complement to this package (Hales *et al.* 2015). *GENETICS* is also publishing “FlyBook” (Cooley *et al.* 2015), a collection of authoritative yet accessible updated summaries on more specific *Drosophila* topics.

A final point is to better engage the large number of researchers who use flies in the laboratory but do not identify primarily as “fly people.” These can include researchers who work with flies alongside other model systems, as well as some who study *Drosophila* neurobiology and behavior. The tremendous growth and depth of *Drosophila* neuroscience, reflected in the focus of the HHMI Janelia Research Campus, has birthed a parallel community with somewhat separate goals, tools, and annual meetings. Increased interaction would benefit all parties.

### Advocacy

Funding is the lifeblood of any field. In the US, most fly research is funded by the NIH. The NIH mission statement includes the following (emphasis ours): “NIH’s mission is to **seek fundamental knowledge about the nature and behavior of living systems** and the application of that knowledge to enhance health, lengthen life, and reduce illness and disability. The goals of the agency are to **foster fundamental creative discoveries, innovative research strategies**, and their applications as a basis for ultimately protecting and improving health…” Even for those who espouse a narrow interpretation of how fundamental research can enhance health, the research occurring in many *Drosophila* labs is clearly aligned with the mission and goals of NIH. With all of the powerful tools and methods available, fly research should be at the heart of the NIH mission.

There has been concern about the impact of NIH funding trends on *Drosophila* research, and on nonvertebrate model organism research in general. To some extent, this reflects the general perception of waning NIH support for investigator-initiated fundamental research as opposed to translational and top-down, “big-science” programs; such concerns have been forcefully expressed in several venues (Alberts *et al.* 2014; Kimble *et al.* 2015; Wangler *et al.* 2015; Spradling 2016). The NIH has responded by explicitly reiterating its support for basic research, emphasizing that “the taxpayer investment in NIH has yielded spectacular returns from basic science over the long term” (Collins *et al.* 2016). It is imperative that this message be received by all study sections and program officers that influence funding decisions.

Objections to *Drosophila* work in some study sections are based on several misperceptions. One of these is ignorance about the fly’s role in discoveries that directly impact human medicine. Many *Drosophila* researchers have encountered their own version of the colleague who asks “Do flies have a Notch gene?” or “…a TRP channel?” This extends to many other conserved molecules and pathways that were first identified and mechanistically elucidated in the fly (Box 1). A related issue is underestimation of the conserved physiology (in addition to molecular genetics) between flies and vertebrates, such as the homologous use of insulin to regulate systemic metabolism despite the fly’s lack of a pancreas (Alfa and Kim 2016). A third misperception is that advancements in genome editing now make vertebrate systems equivalent to the fly for genetic analysis. There is a lack of appreciation of the gulf between even cutting-edge tools for mouse genetics, and those that are routinely used in the fly. Only in the fly can one (for instance) execute genetic mosaics in multiple tissues with fine spatiotemporal control. It is this level of sophisticated *in vivo* manipulation that is often necessary to provide definitive answers to key biological questions.

To make the contributions of fly work clear, the *Drosophila* community should marshal a coordinated effort toward more effective advocacy. For fellow scientists who sit on study sections, review papers, and hire new faculty, reminders of the many precedents of the fly in breaking open new biology (*e.g.*, Box 1) and the definitiveness of *in vivo* analysis can help to correct the misperceptions listed above. For students who are beginning to learn how science works, clear examples of the satisfying answers that emerge from the elegant fly system can spark a lasting appreciation of its value. For the general public, even a passing anecdote about this common insect’s contribution to understanding a human disease can provide a bulwark against antiintellectual attempts to denigrate model organism research. In all these areas, fly workers should keep in mind “the long game,” using education to lay the basis for future support. The fly community can support such advocacy by generating high-quality materials pitched appropriately for each audience; some are already available via the Manchester Fly Facility and others are in the works.

Advocacy can also have more immediate rewards. One recent illustration comes in the context of support to FlyBase. In 2016, changes in funding mechanisms at the NIH brought a proposed reorganization of MODs, including FlyBase, WormBase, the *Saccharomyces* Genome Database, the Zebrafish Information Network, the Mouse Genome Database, and the Rat Genome Database. This reorganization promised increased integration—a sentiment supported by all communities—but also the likely elimination of crucial, species-specific information in the databases, accompanied by a substantial budget cut. FlyBoard considered this a threat to the community’s ability to continue scientific excellence, and initiated a dialogue with other MOD communities to respond. The outcome was a Letter of Support for the MODs, endorsed by a bevy of Nobel laureates, National Academy of Science members, and major scientific societies. When posted online, the MOD support letter drew over 10,000 signatures from >75 countries worldwide. The issue received coverage in major scientific journals (Kaiser 2015; Hayden 2016) as well as extensive social media support. Gratifyingly, Francis Collins acknowledged the letter in an address to The Allied Genetics Conference, saying “Your voice is heard.” A new plan for MOD support is now being worked out under the auspices of The Alliance for Genome Resources. The ability to rapidly mobilize support in a constructive manner that emphasizes the impact of fly research provides an instructive case for community involvement.

### Integration with other research communities

Fly researchers form a strong community, but one that can appear insular to researchers in other fields. Insularity is counterproductive; a tenet of fly work is that lessons learned in this organism are likely to be generally applicable across all animals. To facilitate sharing our discoveries with the broader scientific community, we need to lower the barriers to communication. The ongoing move to integrate MOD databases (see above) aims to create an interface that will allow researchers from other fields to easily access the most relevant information about fly genes and their functions, moving past jargon and species-specific nomenclature. Periodic combined meetings, such as the 2016 Allied Genetics Conference, will also promote information sharing with other genetic model organism communities.

An underexploited connection is with medical researchers and clinicians. The vast knowledge about gene function captured in FlyBase has a tremendous amount to teach researchers working with the complex biology associated with *e.g.*, human genetic variation and its influence on health and therapeutic responses. Recognizing this, NIH has supported core fly resources including the BDSC and FlyBase to expand the availability of strains and information that relate directly to such questions (Millburn *et al.* 2016; Ugur *et al.* 2016). Leveraging these resources is promoted by fly PIs who attend meetings with clinicians or human geneticists, and serve as informal ambassadors to the human biomedical community.

There is growing appreciation of the value of the fly, along with other model organisms, in untangling the complex nature of human disease. Targeted disease classes include neurodegeneration, cancer, and multigenic diseases (Jaiswal *et al.* 2012; Sonoshita and Cagan 2017; Wangler *et al.* 2017). Rare and undiagnosed genetic diseases are another focus, with programs in Canada and the US that match human disease researchers with model organism colleagues to leverage the advantages of model systems for these challenging clinical conditions (https://genematcher.org; https://undiagnosed.hms.harvard.edu). Fly-driven successes in this newly-fledged approach are already emerging (Chao *et al.* 2017; Yoon *et al.* 2017). There are also examples of the fly being used for direct therapeutic screening, at increasing scale, with at least one clinical success providing proof of principle (Vidal 2005; Chang *et al.* 2008; Dar *et al.* 2012; Stickel *et al.* 2015). Though challenges remain, including understanding the basic biology of drug ADME (absorption, distribution, metabolism, and excretion) in flies, an interface with pharmaceutical trials is poised to become a growth area in *Drosophila* research. Similar interfaces with researchers studying other insects as disease vectors, agricultural pests, or pollinators, and as social animals are also exciting opportunities for the *Drosophila* community.

## Summary

We conclude that “the state of the *Drosophila* Research Ecosystem is strong.” Resources, both physical and informational, are outstanding, comprehensive, easily accessible, and growing. Institutions and infrastructure are under effective supervision. Clever tools, including large-scale collections, continue to be developed and freely shared. Leaders carry on the Fly Worker ethos, taking time away from productive research programs to drive community efforts. Most importantly, discoveries published each month demonstrate that the fly is being continuously revitalized in innovative ways to extend its long history of fundamental discovery, as well as to explicitly attack problems of human health and disease.

The engaged Fly community can help maintain and improve its research Ecosystem, and the Fly Board is overseeing a group of initiatives to promote this (Box 4). A clear opportunity exists to create structures that lower barriers for other researchers to leverage *Drosophila* data. This will allow the broader scientific community to realize the full promise of the model organism approach. Finally, we believe that other research groups can learn lessons from the fly community’s experience documented here. As with many ecosystems, *Drosophila* research benefits from active cultivation to ensure a robust, rich, and diverse network.

Box 4 **Initiatives to improve the ecosystem**

The *Drosophila* Board is coordinating the following initiatives with the goal of energizing community members to enhance the *Drosophila* ecosystem.

 Creation of a more comprehensive community contact list, centered around but not limited to registration of fly PIs.

 Community validation of useful reagents, including transgenic RNAi stocks (via RSVP), gRNAs, and commercial  antibodies.

 Encouraging author-initiated annotation of the fly literature (via Fast Track Your Paper).

 Development of standardized reagent forms for fly publications, to promote reproducibility and ease the burden on  FlyBase curators.

 Solicitation of donations of useful stocks and reagents (plasmids and antibodies) to public resource centers, to facilitate the  open sharing and convenient access of reagents.

 A centralized advocacy effort, led by a Fly Board committee, targeted to various audiences.

 Mentoring of new PIs, to promote the successful entry of new labs into the field.

 Promoting fly worker priorities during the creation of the integrated MOD.
